# Role of Homothorax in region specific regulation of *Deformed* in embryonic neuroblasts

**DOI:** 10.1016/j.mod.2015.09.003

**Published:** 2015-11

**Authors:** Raviranjan Kumar, Maheshvari Chotaliya, Sruthakeerthi Vuppala, Ankush Auradkar, Kalyani Palasamudrum, Rohit Joshi

**Affiliations:** a Laboratory of Drosophila Neural Development, Centre for DNA Fingerprinting and Diagnostics (CDFD), 4-1-714, Tuljaguda Complex, Nampally, Hyderabad-500001, India; b Graduate Studies, Manipal University, Manipal 576104, India

**Keywords:** Hox, Deformed, Neural autoregulation, Neuroblast, Neuron, Glia, Homothorax, Homeodomain, Neuromere, Maxillary, Mandibular

## Abstract

The expression and regulation of Hox genes in developing central nervous system (CNS) lack important details like specific cell types where Hox genes are expressed and the transcriptional regulatory players involved in these cells. In this study we have investigated the expression and regulation of *Drosophila* Hox gene *Deformed* (*Dfd*) in specific cell types of embryonic CNS. Using *Dfd neural autoregulatory* enhancer we find that *Dfd* autoregulates itself in cells of mandibular neuromere. We have also investigated the role of a Hox cofactor Homothorax (Hth) for its role in regulating *Dfd* expression in CNS. We find that Hth exhibits a region specific role in controlling the expression of *Dfd*, but has no direct role in mandibular *Dfd neural autoregulatory* circuit. Our results also suggest that homeodomain of Hth is not required for regulating *Dfd* expression in embryonic CNS.

## Highlights

• *Dfd* autoregulates itself in cells of mandibular neuromere in embryonic CNS.
• Hth shows a region specific role in regulating *Dfd* expression in embryonic CNS.
• Hth is critical for *Dfd* expression in neuroblasts of maxillary neuromere.
• Hth is not important for *Dfd* neural autoregulation and its role is restricted to regulating levels of *Dfd*.
• Homeodomain-less isoform Hth is sufficient for its role in *Dfd* regulation in embryonic Nbs.

## 1 Introduction

Hox genes are a highly conserved family of homeodomain containing transcription factors which are well known for their role in specification of the anterior–posterior axis (Pearson et al., 2005). A parallel role for Hox genes in central nervous system (CNS) patterning and development is well known yet not completely understood. Therefore, mechanisms underlying their expression and regulation in CNS need to be investigated. Our understanding of the Hox neural regulation and its functional significance needs further investigation for details like specific cell types where Hox genes are expressed, how they are regulated, their functional significance in those cells, and cell type specific molecular players involved therein.

The phenomenon of autoregulation of Hox genes has been suggested as an important mechanism for their sustained expression during development. To this end both neural and non-neural autoregulatory loops have been identified and investigated in *Drosophila* and vertebrates (Bergson and McGinnis, 1990, Haerry and Gehring, 1996, Kuziora and McGinnis, 1988, Lou et al., 1995, Manzanares et al., 2001, Marty et al., 2001, Muller et al., 1989, Packer et al., 1998, Popperl et al., 1995, Tremml and Bienz, 1992, Yau et al., 2002, Zappavigna et al., 1991).

In *Drosophila*, *Deformed* (*Dfd*), *labial* (*lab*) and *Ultrabithorax* (*Ubx*) are known to autoregulate their expression during development (Bergson and McGinnis, 1990, Kuziora and McGinnis, 1988, Lou et al., 1995, Marty et al., 2001, Muller et al., 1989, Popperl et al., 1995, Tremml and Bienz, 1992). Amongst these three, *Dfd* is known to maintain its expression in both embryonic epidermis and CNS through an autoregulatory transcriptional loop. This autoregulation eventually contributes to the development of maxillary and mandibular segments of the body (Bergson and McGinnis, 1990, Kuziora and McGinnis, 1988, Lou et al., 1995, Muller et al., 1989, Pinsonneault et al., 1997, Popperl et al., 1995, Tremml and Bienz, 1992, Zeng et al., 1994). Hox cofactors Extradenticle (Exd) and Homothorax (Hth) have been shown to play a direct role in maintaining the epidermal autoregulation of *Dfd* in these segments (Bergson and McGinnis, 1990, Joshi et al., 2010, Pinsonneault et al., 1997), but whether they play a similar role in neural autoregulation (Lou et al., 1995) has not been investigated in detail. A role of vertebrate Pbx (Exd homolog) and Meis (Hth homolog) has been shown in Hox neural autoregulation in vertebrate CNS (Manzanares et al., 2001, Popperl et al., 1995), but cell type specific roles of both Pbx and Meis in neural autoregulation have not been studied.

In case of *Dfd* mutants, *Dfd* gene transcription is initiated normally prior to embryonic stage 10. In subsequent stages, *Dfd* mutants are unable to maintain normal *Dfd* expression in both epidermis and CNS, suggesting a role for autoregulation in maintenance of *Dfd* transcription. Previous studies have identified a 3.2 kb intronic enhancer of *Dfd* responsible for its autoregulation in CNS. This enhancer is referred to as *neural autoregulatory enhancer* (*NAE*)*.* A 608 bp fragment of this 3.2 kb successfully recapitulates *Dfd* neural autoregulation. The expression from this enhancer is first detected at stage 11 (~ 5 h after egg laying) in mandibular region of CNS (Lou et al., 1995). The activity of *NAE* is completely abrogated in *Dfd* mutants, thereby making it a good readout for *Dfd* neural autoregulation (Lou et al., 1995, Pinsonneault et al., 1997). The identity of the cells where *NAE* is expressed in CNS and the functional significance of this autoregulation has not been established. While Dfd protein is expressed in both maxillary (Mx) and mandibular (Mn) regions of embryonic CNS, the neural autoregulation has been suggested to be a characteristic of Mn neuromere only. It is known that in *Dfd* mutants there is a loss of expression of *Dfd* in Mn region but a basal level of *Dfd* transcription is still maintained in Mx segments. This suggests that *Dfd* expression in Mx region is independent of neural autoregulation (Zeng et al., 1994). Similarly, in maternal and zygotic mutants of *exd* (*exd*^*mz-*^), Dfd levels and *NAE3.2*-*lacZ* expression in CNS are lowered but qualitative expression of both are essentially unaffected in Mn neuromere (Pinsonneault et al., 1997), thereby suggesting that Exd doesn't play a role in neural autoregulation. A similar role for cofactor Hth has not been checked in embryonic CNS.

In this work, we have investigated the expression and regulation of *Dfd* in specific cell types of embryonic CNS. Our results show that Dfd is expressed in neural stem cells (also called neuroblasts-Nbs), neurons and in glial cells of both Mn and Mx neuromeres. We further report the expression of 3.2 kb *NAE* in all these three cell types of Mn neuromere, thereby suggesting that Dfd autoregulates itself in Mn cells. We have also addressed the role Hth in *Dfd* regulation in embryonic CNS. We find that Hth is critically required for Dfd expression in Nbs of Mx neuromere, while its role in Mn neuromere is limited only in regulating the expression levels of Dfd in these cells, and has no function in neural autoregulatory circuit. Our experiments further suggest that homeodomain of Hth is not necessary for *Dfd* regulation, and HD-less form of Hth is sufficient for *Dfd* regulation in embryonic Nbs.

## 2 Results

The current work focuses on identifying specific cell types of CNS where *Dfd* is expressed and autoregulated; and to understand the role of Hth in regulating Dfd expression in embryonic Nbs.

### 2.1 Region specific expression analysis of 3.2 kb *NAE* in embryonic CNS

*Dfd* autoregulation in embryonic CNS is mediated through a 3.2 kb *neural autoregulatory enhancer* (*NAE3.2*) (Lou et al., 1995, Pinsonneault et al., 1997, Zeng et al., 1994). This enhancer primarily expresses in Mn neuromere and loses its CNS specific expression in *Dfd* mutants (Lou et al., 1995, Pinsonneault et al., 1997, Zeng et al., 1994), making *NAE3.2-lacZ* a bona fide readout for neural autoregulation.

**Fig. 1:**
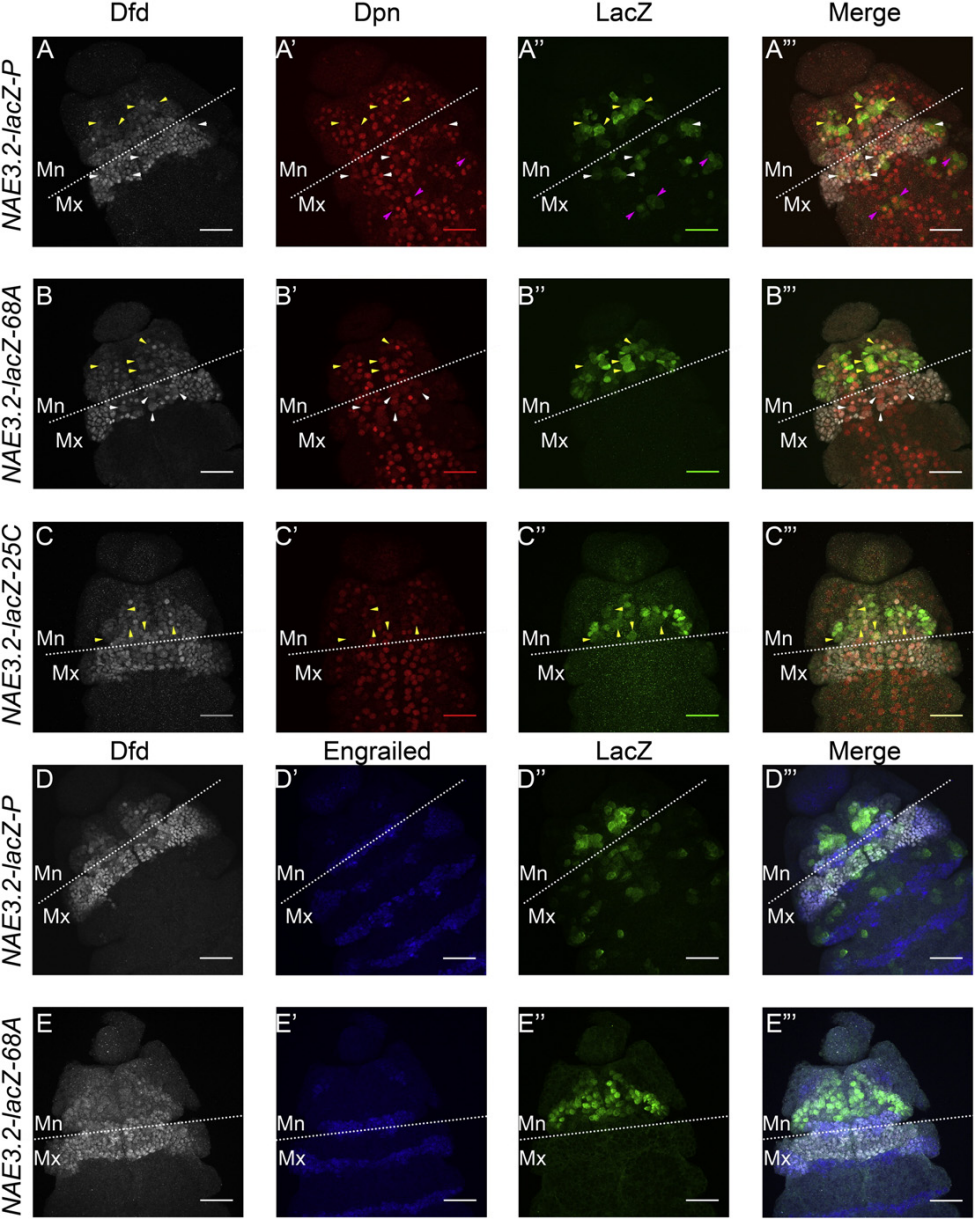
Comparative expression analysis of *NAE3.2-lacZ-P* with *NAE3.2-lacZ-68A* and *25C.* Stage 12 embryos from the three transgenic lines costained with Dfd, LacZ and Nb specific marker Dpn (Panels-A, B and C) are shown. Expression of *NAE3.2-lacZ-P* in Dpn positive cells of more posterior segments outside Dfd region is shown by pink arrowheads (Panel A”). Nbs in Mx segment coexpressing Dfd and lacZ for *NAE3.2-lacZ-P* line are shown by white arrowheads (Panel A). No LacZ coexpression is seen in Mx Nbs in case of *NAE3.2-lacZ-68A* and *25C* lines (white arrowheads, Panels B and C). A comparison stage 12 embryos costained with Dfd, lacZ and segmentation marker Engrailed (Panels-D and E) are shown. The expression of *NAE3.2-lacZ-P* (Panel D) and *NAE3.2-lacZ-68A* (Panel E) in Mn and Mx neuromeres is shown by costaining for Dfd, En and LacZ. No lacZ expression is seen in Mx neuromere in both *68A* and *25C* lines. Mn and Mx segment boundary is indicated by white dotted line and is also marked by En staining in panels D and E. Yellow arrowheads indicate the cells with coexpression of Dfd, LacZ and Dpn, while white arrowheads indicate the cells which coexpress Dfd and Dpn. Pink arrowheads show the cells which are coexpressing Dpn and LacZ but are Dfd negative. Scale bars shown are for 30 μm.

We started out by looking at the expression of *NAE3.2-lacZ* line reported earlier (obtained from McGinnis lab-UCSD, referred to as *NAE3.2-lacZ-P* in the text and figures) (Lou et al., 1995, Pinsonneault et al., 1997). A costaining of β-galactosidase (LacZ), Dfd and Dpn (Nb marker) on embryos of *NAE3.2-lacZ-P* showed the expression of LacZ outside Dfd specific region of CNS (Fig. 1A–A”, pink arrow heads, Z-project of multiple slices is shown). Since earlier results had suggested that Dfd maintains its expression through neural autoregulation primarily in Mn neuromere, we generated and analyzed additional reporter lines and compared their expression to *NAE3.2-lacZ-P*. These lines were generated by site specific insertion (Bischof et al., 2007) of *NAE3.2-lacZ* constructs at attP2-*68A* and attP40-*25C6* (and will here on be referred to as *NAE3.2-lacZ-68A* and *25C*)*.*

**Supplementary Fig. 1:**
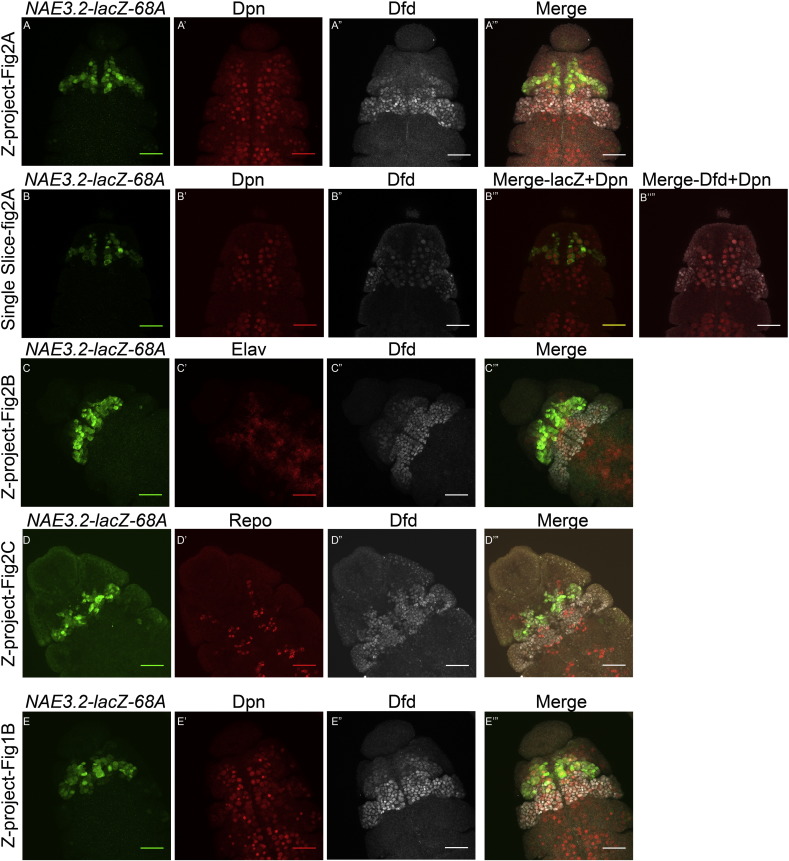
Two channel merge and channel Z-projects of selected embryos. Z project of the embryos (shown in Fig. 2) stained with Dfd, lacZ and cell specific marker is shown in Panels A, C and D. Panel B shows two channel merge for the single slice for Dpn, lacZ and Dfd stained embryos shown in Fig. 2A. Panel E shows Z-project for the Dpn, lacZ and Dfd stained embryos shown in Fig. 1B.

In case of both lines (*NAE3.2-lacZ-68A* and *25C*), we observed that LacZ reporter expression was confined primarily to Dfd region (Fig. 1B–B” and C–C”) and very little background was observed outside Dfd region (Z-project of multiple slices is shown in Fig. 1; for complete Z project of Fig. 1B, see Supp. Fig. 1E). It was also observed that the expression of *NAE3.2-lacZ-68A* and *25C* was mainly confined to Mn neuromere of CNS and there were only very few cells of Mx neuromere (outside of CNS) which showed LacZ expression compared to *NAE3.2-lacZ-P* (Fig. 1B–B” and C–C”). In order to further clarify the region specific expression of *NAE3.2-lacZ* reporter, a costaining for Dfd, Engrailed and LacZ antibodies was done for both *NAE3.2-lacZ-P* and *NAE3.2-lacZ-68A*. It was observed that while the expression of *NAE3.2-lacZ-P* extended into Mx neuromere and cells outside Dfd region (Fig. 1D–D’”), LacZ expression in *NAE3.2-lacZ-68A* was confined to Mn neuromere of the embryo (Fig. 1E–E’”).

These results suggest that *NAE3.2-lacZ* reporter is primarily confined to Mn neuromere (in both *68A* and *25C* lines) and thus lines could be used as an accurate readout for neural autoregulation in this region. It further suggests that the expression of *NAE3.2-lacZ-P* in regions other than Mn neuromere may not have any functional significance. Thus all subsequent experiments were done with *NAE3.2-lacZ-68A* reporter line.

### 2.2 Dfd is expressed and autoregulated in mandibular Nbs, neurons and glial cells

**Supplementary Fig. 2:**
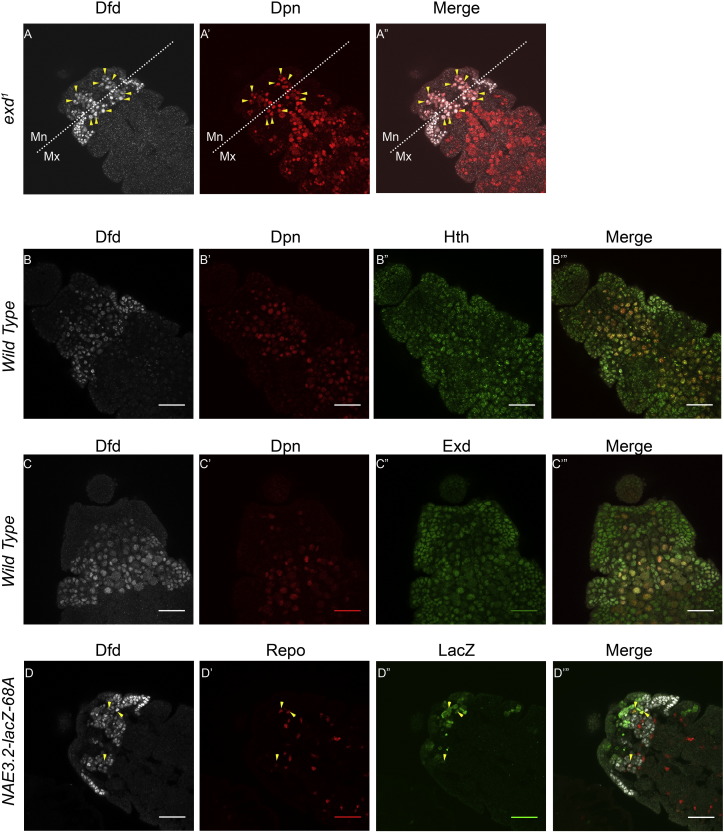
Exd and Hth express in embryonic Nbs. Embryos costained with different antibodies are shown. Antibodies used are shown at the top of the panels, genotypes are shown on the left. All embryos are stage 12 except for Panel D which is a stage 13 embryo. Yellow arrowheads in panel A indicate the cells which coexpress Dfd and Dpn in *exd*^*1*^ mutants. Yellow cells in B”’ and C”’ are cell coexpressing Dfd/Dpn/Hth and Dfd/Dpn/Exd respectively. Yellow arrowheads in Panel D show glial cells in stage 13 embryos coexpressing *NAE3.2-lacZ* and Dfd.

**Fig. 2:**
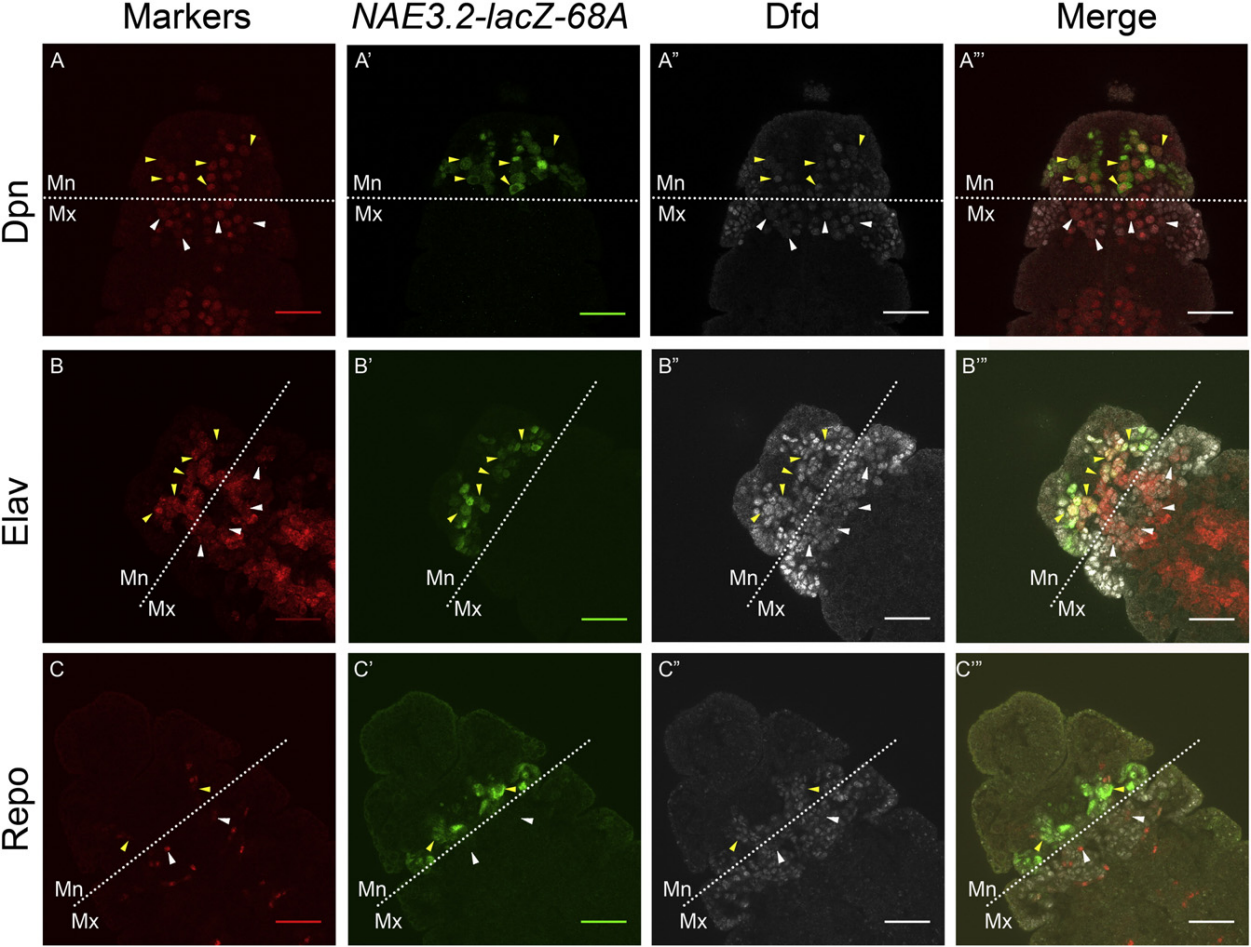
*Dfd* expression and autoregulation in Nbs and neurons and glial cells. Stage 12 embryos from *NAE3.2-lacZ-68A* line costained with Dfd, LacZ and cell specific marker are shown, Dpn (Panel-A), Elav (Panel-B) and Repo (Panel-C). Dfd is autoregulated through 3.2 kb *NAE* in Nbs, neuron and glial cells of Mn neuromere, while its expression in cells of Mx region is not autoregulated. Mn and Mx segment boundary is indicated by white dotted line, yellow arrowheads indicate Mn cells with coexpression of Dfd, LacZ and cell specific markers, while white arrowheads indicate Mx cells which coexpress Dfd and cell specific marker only and are lacZ negative. Scale bars shown are for 30 μm.

In order to identify the specific cell types where Dfd is expressed and autoregulated in embryonic CNS, a costaining for Dfd protein was done with LacZ, and Nb (Dpn), neuron (Elav) and glial cell (Repo) specific markers. We found that Dfd protein was expressed in all the Nbs (yellow and white arrowhead in Fig. 2A–A”’, see Supp. Fig. 1B for two channel merges) and most of the neurons (yellow and white arrowhead in Fig. 2B–B’”) of Mn and Mx neuromeres of CNS at stage 12 of embryogenesis. We observed that there were few glial cells present at stage 12 of embryogenesis (yellow and white arrowhead in Fig. 2C–C”’) and all of them express Dfd at a very low level. More glial cells were observed in later stages of embryonic development (stages 13 and 14) and Dfd expression was found to be more robust in the later stages (stage 13 embryos shown in Supp. Fig. 2D).

To further test if the expression of Dfd in Nbs, neurons and glial cells in Mn neuromere was maintained through autoregulation, we looked at the expression of *NAE3.2-lacZ* and Dfd in these cells. Our results showed that LacZ coexpressed with Dfd in Nbs (yellow arrowheads, Fig. 2A–A”’); neurons (yellow arrowheads Fig. 2B–B’”); and glial cells (yellow arrowheads Fig. 2C–C’”). Glial cells showed a very weak expression of LacZ and Dfd (yellow arrowheads Fig. 2C–C”’) at stage 12, but the expression of Dfd and LacZ became stronger in subsequent embryonic stages (Supp. Fig. 2D).

These results indicate that Dfd is expressed in all the three cell types of CNS (Nbs neurons and glial cells) in both Mx and Mn neuromeres. The coexpression of *NAE3.2-lacZ* in Mn cells further suggests that Dfd expression in these cells is autoregulated, while in Mx cells Dfd is expressed but not autoregulated. Since Nbs are neural progenitor cells (that give rise to all the cells of CNS including neurons and glial cells), we decided to restrict our subsequent analysis to Nbs only.

### 2.3 Hth shows a region specific effect on *Dfd* expression in Nbs

Hox genes function with cofactors like Exd and Hth (Pearson et al., 2005), which have been shown to play an important role in non-neural autoregulatory loops for *Dfd* and *labial* (Bergson and McGinnis, 1990, Joshi et al., 2010, Kuziora and McGinnis, 1988, Lou et al., 1995, Marty et al., 2001, Popperl et al., 1995, Tremml and Bienz, 1992). While the role of *exd* has been tested in *Dfd* neural autoregulation (Pinsonneault et al., 1997), a similar role for Hth has not been investigated.

**Fig. 3:**
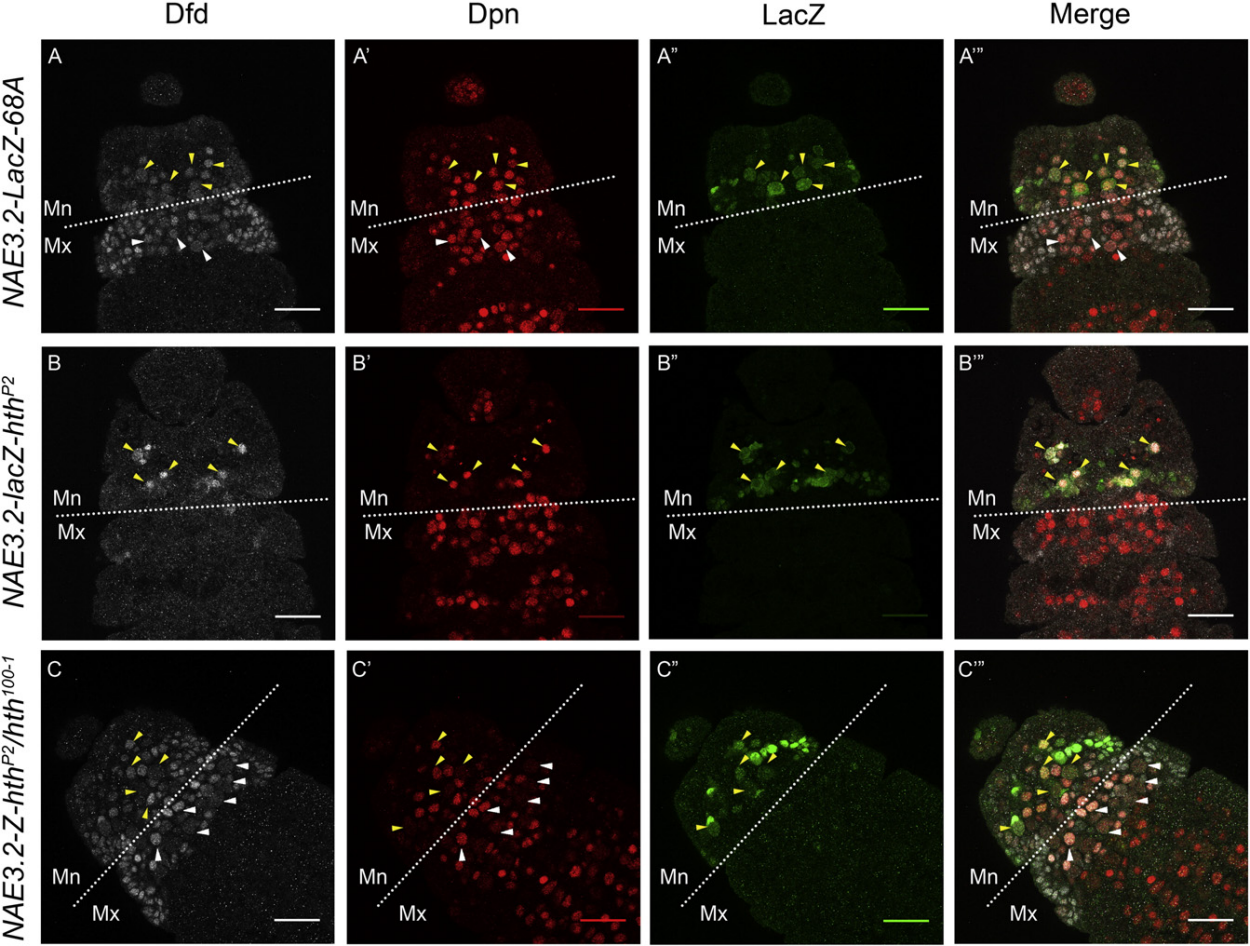
Region specific role of Hth in regulating Dfd expression in Nbs. Stage 12 embryos of the following genotypes costained with Dfd, Dpn and LacZ are shown, *wild type* (Panel-A), *hth*^*P2*^ (Panel-B) and HM-Hth expressing embryos (Panel-C, embryos of genotype *hth*^*P2*^/*hth*^*100-1*^ express only the HD-less form of Hth, HM-Hth). *hth*^*P2*^ mutant embryos only show a Mx Nbs specific loss of Dfd expression and a dramatic decrease in level of Dfd in Mn Nbs (yellow arrowheads in Panel B). The *NAE3.2-lacZ* expression in Mn NBs is unaffected in *hth*^*P2*^ mutant embryos (yellow arrow head in Panel-B”). The brightness of the Dfd channel in Panel B has been increased to clearly show the residual expression of Dfd in Nbs of Mn neuromere. Mn and Mx segment boundary is indicated by white dotted line. Yellow arrowheads indicate Mn cells with coexpression of Dfd, Dpn and lacZ, while white arrowheads indicate Mx cells which coexpress Dfd and Dpn only and are lacZ negative. Scale bars shown are for 30 μm.

To this end, we decided to start with *exd*^*1*^ mutant. *exd*^*1*^ homozygous mutants (Peifer and Wieschaus, 1990) showed no significant change in Dfd expression in Nbs, both in Mx and Mn neuromeres (yellow arrowhead Supp. Fig. 2A). This is because Exd is known to be maternally contributed (Peifer and Wieschaus, 1990, Pinsonneault et al., 1997). Since Hth is a known partner of Exd, and plays an important role in its transport into the cell nuclei (Kurant et al., 2001), we next looked at *hth* mutant. We expected that *hth* null mutant will mimic a phenotype similar to *exd* complete loss of function (Kurant et al., 2001, Rieckhof et al., 1997). Our analysis of *hth* null mutant (*hth*^*P2*^) (Rieckhof et al., 1997), showed almost complete absence of epidermal Dfd expression in embryos (data not shown). Interestingly, we observed a region specific effect of *hth* mutation on Dfd expression. We found that Dfd expression was completely missing in Mx Nbs (Fig. 3, Panel B), while the expression in Mn Nbs was dramatically down regulated, but low levels of Dfd could still be observed in these cells (yellow arrow heads, Fig. 3, Panel B). The brightness of the Dfd channel in Fig. 3, Panel B has been increased to highlight the residual expression of Dfd in Nbs of Mn neuromere. Furthermore the expression of *NAE3.2-lacZ* was largely unaffected in the Mn Nbs (Fig. 3, Panel-B”), suggesting that *Dfd* autoregulatory transcriptional loop is unaffected in *hth*^*P2*^ mutants. This result is very similar to what was observed in the past for *exd*^*mz-*^ mutant embryos (embryos mutant for maternal and zygotic *exd*). In these mutants only a quantitative decrease in expression of Dfd was observed. The expression of *NAE3.2-lacZ* showed a slight decrease in Mn neuromere, but the qualitative expression of both Dfd and *NAE3.2-lacZ* was unaffected in *exd*^*mz-*^ mutant (Pinsonneault et al., 1997).

These results suggest that Hth plays an important region specific role in regulation of *Dfd* in Nbs of embryonic CNS. We find that Hth is critical for Dfd expression in Mx Nbs but is important only for maintenance of the levels of Dfd protein in Mn Nbs, and has no role in *Dfd* neural autoregulation.

### 2.4 Dfd-Exd-HthFL bind as a trimer on *NAE*

**Fig. 4:**
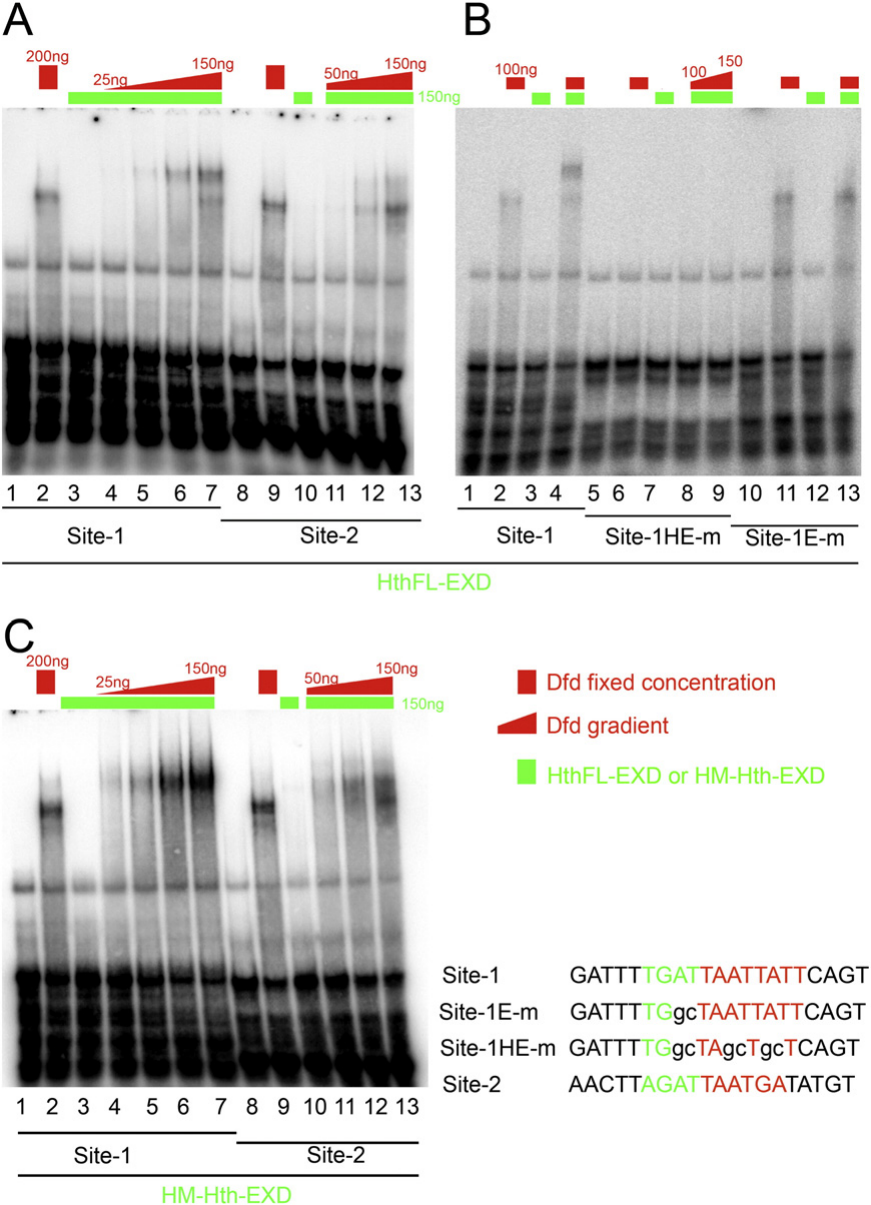
Hox, Exd and Hth bind to sites-1 and 2 of *NAE*. Panel-A shows that Exd-HthFL along with Dfd show binding on sites-1 and 2. Panel-B shows that site-1 probe mutant for Exd binding site loose cooperative binding of Dfd-Exd-HthFL trimer, while or site-1 probe mutant for Hox-Exd binding site shows a complete loss of Dfd-Exd-HthFL binding. Panel-C shows that HM-Hth promotes binding of Dfd on sites-1 and 2. A constant concentration of 150 ng per reaction is used for both Exd-HthFL and Exd-HM-Hth, indicated by a green box of uniform height. Constant concentrations (of 100 and 200 ng) of Dfd wherever used are indicated by a red box (of X and 2X height). Varying concentration of Dfd is indicated by right triangle with lowest and highest concentrations indicated (25, 50, 100 and 150 ng of Dfd protein were used in lanes 4 to 7 and 50, 100 and 150 ng of Dfd were used in lanes 11 to 13 of Panels A and C). Wild type sequence for sites-1 and 2 is shown along with Exd mutant (site-1-E-m) and Hox-Exd mutant (site-1-HE-m) version of site-1. Mutation in site-1 is indicated in lower case. Hox and Exd binding halves on these binding sites are indicated by red and green color of the text.

A general decrease in levels of Dfd was observed in Mn neuromeres in case of both *exd* (*exd*^*mz-*^) (Pinsonneault et al., 1997) and *hth* mutants (Section 2.3). This suggests that both these factors play an important role in controlling the levels of Dfd in Mn neuromere. Since Mn expression of Dfd is regulated through 3.2 kb *NAE*, we decided to test the Dfd binding in the presence of Hth and Exd on Hox-Exd composite binding sites present in 3.2 kb *NAE.* The 3.2 kb *NAE* enhancer has seven composite Hox-Exd binding sites (with consensus sequence of [T/A]GATNNATNN). We checked all these 7 binding sites for Dfd-Exd-Hth binding by EMSA. Two out of these seven sites are also found in 608 bp *NAE* which is known to recapitulate the expression of 3.2 kb enhancer (Lou et al., 1995). The binding data for these two sites (sites-1 and 2) has been presented in Fig. 4. We tested the role of Exd and HthFL (Hth Full length) heterodimer along with Dfd for their capacity to bind on sites-1 and 2 by EMSA. We found that while Dfd protein bound to both of the binding sites (Fig. 4, Panel A, lanes 2 and 9); co-purified Exd-HthFL heterodimer didn't show any significant binding on its own to either of the binding sites (Fig. 4, Panel A, lanes 3 and 10). A Dfd-Exd-HthFL trimer showed cooperative binding on site-1 (Fig. 4, Panel A, lanes 4 to 7) while on site-2 it showed comparatively weaker trimer binding (Fig. 4, Panel A, lanes 11 to 13). The site-1 oligo mutant for Exd binding site alone showed a loss of cooperative binding and only Dfd monomer bound to DNA (Fig. 4, Panel B, lane 13), suggesting that cooperative trimer binding seen on site-1 is due to Exd-HthFL. The oligo mutant for both Hox-Exd binding site showed a complete loss of binding for Dfd monomer as well as for Dfd-Exd-HthFL trimer (Fig. 4, Panel B, lanes 6, 8 and 9). These results show that Dfd forms a cooperative trimer with Exd-HthFL *in vitro* on *NAE*.

### 2.5 Homeodomain-less isoform of Hth is necessary for its role in *Dfd* regulation in embryonic Nbs

Homeodomain-less (HD-less) isoform of Hth (referred to as HM-Hth) has been shown to be a functionally important isoform in embryonic stages of development (Noro et al., 2006). In order to test if Dfd expression (in both Mn and Mx neuromeres) is dependent on full length Hth or HM-Hth, we analyzed embryos which expressed only HM-Hth isoform (*hth*^*P2*^/*hth*^*100-1*^ heteroallelic combination (Noro et al., 2006)). We observed that HM-Hth embryos showed normal expression of Dfd in Nbs of both Mx and Mn neuromeres of embryonic CNS (yellow and white arrowheads, Fig. 3, Panel C). *NAE3.2-lacZ* expression in Mn neuromere was also unaffected in the embryos expressing only HM-Hth isoform (yellow arrowheads, Fig. 3, Panel C”). In order to test the role of HM-Hth and Exd for their capacity to form a trimer complex with Dfd on Hox-Exd composite binding sites, we tested HM-Hth-Exd heterodimer and Dfd binding on sites-1 and 2 by EMSA. In concurrence to our *in vivo* results we observed that Dfd-Exd-HM-Hth showed a cooperative binding on both of the binding sites (Fig. 4 Panel C, lanes 4–7 and lanes 11–13). The trimer binding was highly cooperative on site-1 and was comparatively less cooperative on site-2 (Fig. 4, compare lanes 4–7 and 11–13 for Panels A and C). Thus, our *in vivo* experiments suggest that in Mn neuromere HM-Hth is sufficient for maintaining *Dfd* expression levels (probably through its participation with Exd – see discussion – Section 3.4). While our *in vitro* results suggest that HM-Hth is sufficient to interact with Exd and Dfd to assemble a cooperative trimer complex on *NAE3.2*. This trimer complex may have a role in maintaining Dfd expression levels in Mn region. Additionally, the expression of Dfd in Mx neuromere requires only HM-Hth (and Exd). The fact that Hth is sufficient to carry out its requisite role in both the neuromeres without its HD, suggests that HD of Hth is not necessary for region specific role of Hth in CNS.

## 3 Discussion

Our understanding of expression and regulation of Hox genes in embryonic CNS has been lacking important details like specific cell types where Hox genes are expressed and the regulatory molecular players involved in these cells. In *Drosophila*, *Dfd* autoregulation has been investigated and established in both epidermal and neural cells. Specific enhancers have been isolated which control Dfd expression in both these tissues (Bergson and McGinnis, 1990, Kuziora and McGinnis, 1988, Lou et al., 1995). Therefore comparison of Dfd expression (in specific cell types) to 3.2 kb *Neuro Autoregulatory Enhancer* (*NAE*) expression gave us an opportunity to understand the role of Hth in Dfd expression and autoregulation.

### 3.1 3.2 kb *Neuro Autoregulatory Enhancer*

*Dfd* autoregulation happens in Mn neuromere of CNS through a 3.2 kb *NAE*, which critically depends on Dfd protein for its expression in cells of CNS (Lou et al., 1995, Zeng et al., 1994). Our analysis of the original *NAE3.2-lacZ-P* line (obtained from McGinnis lab) showed us a strong expression of LacZ in Nbs and neurons within (Fig. 1 Panel A, yellow and white arrowheads) and outside the region of Dfd expression (Fig. 1 Panel A, pink arrowheads). Since earlier results had suggested that Dfd maintains its expression through neural autoregulation primarily in Mn neuromere our analysis of *NAE3.2-lacZ-P* line led us to generate and analyze new reporter lines for 3.2 kb *NAE* by site specific insertion (Bischof et al., 2007). In comparison to *NAE3.2-lacZ-P* line both *NAE3.2-lacZ-68A* and *25C* lines showed us expression only in Dfd specific region of embryonic CNS. The specificity of *NAE3.2-lacZ-68A* expression within Dfd region was further established by a costaining of Dfd, LacZ and Engrailed (Fig. 1 Panel E). These results suggested that 3.2 kb *NAE* shows a very specific expression mainly confined to Mn neuromere of embryonic CNS, with a very minor expression in maxillary region. These results reconfirmed that *Dfd* neural autoregulation happens mainly in Mn neuromere of embryonic CNS through 3.2 kb *NAE*. Based on these results we suggest that the expression of *NAE3.2-lacZ-P* outside Mn neuromere may not be functionally significant.

### 3.2 *Dfd* autoregulates itself in mandibular Nbs neurons and glial cells

Using antibodies to cell type specific markers, we found that Dfd is expressed in embryonic Nbs, neurons and glial cells (Fig. 1). Since *NAE3.2-lacZ* is an established read out of *Dfd* neural autoregulation in Mn neuromere, the coexpression of Dfd and *NAE3.2-lacZ* in Nbs, neurons and glial cells suggest that *Dfd* autoregulates itself through *NAE3.2* in these cells. Our analysis of Mn neurons indicated that there were few neurons which were Dfd^+^/Elav^+^/lacZ^−^, we speculate that LacZ expression in these cells was below our detection limit.

The expression of Dfd in glial cells was analyzed in stages 12 (Fig. 2 Panel C) and 13 (Supp Fig. 2D). We found few glial cells at early stage 12 (Fig. 2 Panel C), this probably was because majority of glial cells were yet to be born. Both *NAE3.2-lacZ* and Dfd also showed a very weak expression in glial cells initially. As the development progress we find more number of glial cells, and expression of Dfd and *NAE3.2-lacZ* becomes stronger and consistent in these cells (Stage 13 embryo, Supp Fig. 2D). As expected Mx glial cells expressed Dfd but not LacZ (white arrowheads in Fig. 1, Panel C).

Since Nbs, neurons and glial cells in Mx neuromere do not autoregulate Dfd expression, it will be of future interest to investigate if these cells sustain Dfd expression later in development.

### 3.3 Segment specific role of Hth in Dfd regulation

Exd is known to play a role in *Dfd* autoregulation in epidermis (Bergson and McGinnis, 1990), but neural autoregulation had been shown to be independent of Exd (Pinsonneault et al., 1997). Dfd expression in maternal-zygotic *exd* mutant embryos (*exd*^*mz-*^) showed a significant decrease compared to the controls, but more importantly both Dfd and *NAE3.2-lacZ* showed only a quantitative decrease in expression while the qualitative expression was unaffected (Pinsonneault et al., 1997). Our results with *exd*^*1*^ mutant showed us no significant change in expression of Dfd in embryonic Nbs (Supp. Fig. 2A), this was due to maternal contribution of Exd. On the other hand, in *hth*^*P2*^ mutants, we find that the expression of *NAE3.2-lacZ* is unaffected in Mn Nbs but the expression of Dfd in these cells is dramatically reduced. This is in addition to complete loss of Dfd expression from Mx Nbs. This data suggests a region specific role of Hth in regulation of Dfd expression in embryonic Nbs. We conclude that Hth is critically required for the expression of Dfd in Nbs of Mx neuromere, whereas its role in Mn Nbs is limited only in regulating the expression levels of *Dfd*. These observations further suggest that Hth has no role to play in regulation of core neural autoregulatory circuit. This is very similar to what was observed in the past for *exd*^*mz-*^ mutant embryos (Pinsonneault et al., 1997), therefore it further supports the idea that Exd and Hth could be functioning together in Nbs of both Mx and Mn neuromeres. This is relevant since Hth is known to play an important role in nuclear localization of Exd (Kurant et al., 2001, Noro et al., 2006). We speculate that in case of *hth*^*P2*^ mutants, Exd is not be able to localize to nucleus and thus cannot carry out its role in regulating *Dfd* expression in Mx cells. In Mn neuromere on the other hand Exd-Hth heterodimer may have a role only in maintaining the levels of *Dfd* expression.

The expression of Dfd in Mn Nbs is autoregulated through *NAE3.2.* Our results suggest that Exd-Hth heterodimer plays a role in maintenance of the levels of Dfd in these cells. By this argument, it is expected that expression of *NAE3.2-lacZ* should decrease in Mn Nbs in case of both *exd*^*mz-*^ and *hth*^*P2*^ mutants. Interestingly in both these cases *NAE3.2-lacZ* levels show a very minor decrease, while Dfd protein levels show a dramatic decrease. There can be two explanations for these observations; first one being that the enhancer for maintenance of the levels of Dfd protein lies outside *NAE3.2* or *NAE3.2* has two modules, one of which is responsible for the autoregulation and other one is important for the maintenance of the *Dfd* expression levels. The latter line of thinking is further corroborated by a very low expression of LacZ reporter when a 608 bp sub-fragment of 3.2 kb *NAE* is used to drive LacZ *in vivo* (Lou et al., 1995). Therefore it is plausible that Dfd-Exd-Hth may play a role in enhancing the mandibular levels of Dfd through one of these 7 composite Hox-Exd binding sites found in *NAE.*

Furthermore the fact that *NAE3.2-lacZ* expression is not affected much in *exd*^*mz-*^ and *hth*^*P2*^ mutants (as compared to dramatic decrease of Dfd expression) could be attributed to the universal heat shock promoter used in *NAE3.2-lacZ* construct, which may make the construct less sensitive towards Exd-Hth mediated expression level control (Lorberbaum and Barolo, 2015, Zabidi et al., 2015). Replacing the universal promoter with endogenous Dfd promoter can be done to address this issue.

### 3.4 Role of HM-Hth in *Dfd* autoregulation

Our results also show that HD of Hth is not necessary in *Dfd* regulation in embryonic Nbs. We find that Mx and Mn expressions (as well as Mn autoregulation) of Dfd is unaffected in embryos expressing only HD-less isoform of Hth (HM-Hth; only isoform expressed in *hth*^*P2*^/*hth*^*100-1*^ embryos (Noro et al., 2006)). HM-Hth is suggested to interact with Exd and promotes its nuclear localization normally (Kurant et al., 2001, Noro et al., 2006). Our EMSA results show that HM-Hth along with Exd was able to form a cooperative trimer (Dfd-Exd-HM-Hth) on both sites-1 and 2 (Fig. 4, Panel C). In fact, our results indicate that Exd-HM-Hth-Dfd trimer on DNA is much more cooperative than Dfd-Exd-HthFL on both these sites (compare lanes 4–7 and 11–13 in Panels A and C of Fig. 4, which used the same concentrations of Dfd, HthFL-Exd and HM-Hth-Exd). The results with *hth*^*P2*^/*hth*^*100-1*^ embryos also suggest that HM-Hth may contribute to Mx and Mn expressions of Dfd primarily with the help of Exd protein. Therefore taking into account our *in vitro* and *in vivo* results, we like to speculate that HM-Hth has a limited role along with Exd and Dfd in regulating *Dfd* levels in Mn Nbs, and HM-Hth doesn't affect *Dfd* neural autoregulation in these cells. On the other hand we speculate a direct role of Exd-HM-Hth in regulating *Dfd* expression in Mx neuromere. The role of Exd in Mx Nbs could be tested either by attempting Nb specific RNA interference or by making germline clones of *exd.*

Since both Exd and HM-Hth are required only for regulating levels of Dfd expression in mandibular Nbs, and neural autoregulation in these cells is independent of their roles, therefore we propose a role for yet to be identified factor(s) in regulating core neural autoregulatory transcriptional loop.

Identification of this/these factor(s) and characterization of their role in Nbs and differentiated neurons of mandibular region will be an interesting direction for future research.

## 4 Experimental procedure

### 4.1 *Drosophila* strains and genetics

Wild type flies used were *w1118*. Standard methods were used to recombine and balance the chromosome containing mutation and transgenes. The *hth*^*P2*^ mutation and *exd*^*1*^ mutations (Peifer and Wieschaus, 1990, Rieckhof et al., 1997) were balanced over *hb-lacZ*-marked *TM3-Sb* balancer and *ftz-lacZ* marked *FM7* balancer to identify homozygous embryos. The *hth*^*100-1*^mutation (Noro et al., 2006) was balanced over *TM3-Sb* balancer marked with KrGAL4, UAS-GFP transgene. *NAE3.2-lacZ* transgenic line was made using the phiC31-based integration system (Bischof et al., 2007) and inserted in attP sites at *68A4* and *25C6*. The original *NAE3.2-lacZ* line (generated using classical P-element based transgenic method) is balanced on X chromosome and was obtained from Prof. W. McGinnis (UCSD) and referred to as *NAE3.2-lacZ-P* everywhere. All the experiments were done with 4-h egg collections which were aged for 6 h at 25 °C.

### 4.2 Antibodies and immunohistochemistry

Antibody staining was done as previously described (Noro et al., 2006). The following primary antibodies were used: anti-lacZ (Chicken, AbCam-ab9631, 1:2000), anti-Dfd (Rbt, Preabsorbed, 1:500), anti-Dpn (mouse, Preabsorbed, 1:1000) anti-Repo (mouse, DHSB 8D12, 1:100), anti-Elav (rat, DSHB, 7E8A10, 1:100), anti-Exd (mouse, DSHB, 1:5), anti-Hth (guinea pig, 1:500) and anti-En (mouse, DSHB, 4D9, 1:50). Secondary antibodies conjugated to Alexa fluorophores from Molecular Probes were used, dilution used is in parenthesis: AlexaFluor405 (1:200), AlexaFluor488 (1:500), AlexaFluor555 (1:1000), and AlexaFluor647 (1:500). Embryos were mounted with Vectashield. Z-series images were collected on Zeiss LSM700 and were analyzed by LSM browser and ImageJ (http://rsbweb.nih.gov/ij). Other image analyses were done with Photoshop CS3. All images were acquired at 63 × and 0.5 zoom. All the analysis represented in the figure was done on stage 12 embryos. All figures unless specified were single confocal slices of 0.4 μm thickness, Fig. 1 was Z-project of multiple slices. All images have anterior roughly towards the top (or top left corner) and posterior towards the bottom (or bottom right corner). Scale bars shown are for 30 μm.

### 4.3 Protein-DNA binding assay

Site-1 and site-2 were examined by EMSA for protein binding.

Sequence of all oligos used for making probes are given below, the specific binding site tested is underlined.

Site-1 (wild type).

ctgacatcctaacagttgcgcgccatttgattttgattaattattcagtAGCTGTGGGACGAGG.

Site-1Em (mutant for Exd binding site).

ctgGcGCcctaacagttgcgcgccatttgattttgGCtaattattcagtAGCTGTGGGACGAGG.

Site-1HE-m (mutant for Hox-Exd binding site).

ctgacatcctaacagttgcgcgccatttgattttgGCtaGCtGCtcagtAGCTGTGGGACGAGG.

Site-2.

tgggggcctgtcaacggttggcttgacacatatcattaatctaagtttcAGCTGTGGGACGAGG.

EMSA was carried out as described previously (Joshi et al., 2010).

All proteins were purified using 6XHis tag in the N-terminal of the protein. Full-length Exd, was copurified with the HM domain of Hth (or with full length Hth, HthFL) from *Escherichia coli* and copurified protein was used at 150 ng per reaction. Dfd protein used in experiments was a truncated form (residues 130–586) of DfdWT protein (Joshi et al., 2010).

The following are the supplementary data related to this article.

**Supplementary Fig. 3:**
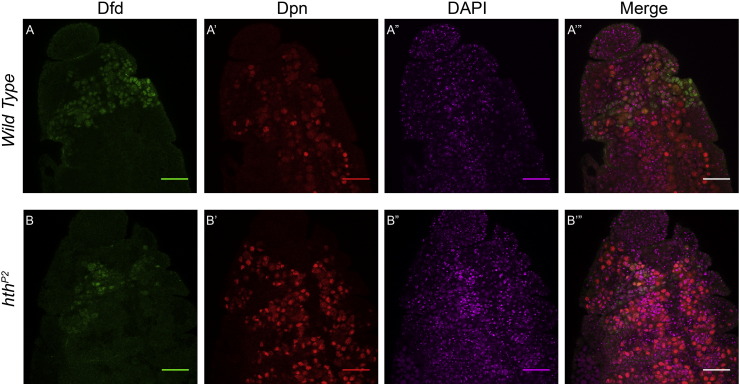
DAPI staining for control and *hth*^*P2*^ mutants. Single slice of stage 12 embryos of control and *hth*^*P2*^ mutants costained with Dfd, Dpn and DAPI is shown. The number of nuclei marked by DAPI in control and *hth*^*P2*^ mutants do not show any significant difference.

## References

[bb0005] Bergson C., McGinnis W. (1990). An autoregulatory enhancer element of the *Drosophila* homeotic gene Deformed. EMBO J..

[bb0010] Bischof J., Maeda R.K., Hediger M., Karch F., Basler K. (2007). An optimized transgenesis system for *Drosophila* using germ-line-specific phiC31 integrases. Proc. Natl. Acad. Sci. U. S. A..

[bb0015] Haerry T.E., Gehring W.J. (1996). Intron of the mouse Hoxa-7 gene contains conserved homeodomain binding sites that can function as an enhancer element in *Drosophila*. Proc. Natl. Acad. Sci. U. S. A..

[bb0020] Joshi R., Sun L., Mann R. (2010). Dissecting the functional specificities of two Hox proteins. Genes Dev..

[bb0025] Kurant E., Eytan D., Salzberg A. (2001). Mutational analysis of the *Drosophila* homothorax gene. Genetics.

[bb0030] Kuziora M.A., McGinnis W. (1988). Autoregulation of a *Drosophila* homeotic selector gene. Cell.

[bb0035] Lorberbaum D.S., Barolo S. (2015). Enhancers: holding out for the right promoter. Curr. Biol..

[bb0040] Lou L., Bergson C., McGinnis W. (1995). Deformed expression in the *Drosophila* central nervous system is controlled by an autoactivated intronic enhancer. Nucleic Acids Res..

[bb0045] Manzanares M., Bel-Vialar S., Ariza-McNaughton L., Ferretti E., Marshall H., Maconochie M.M., Blasi F., Krumlauf R. (2001). Independent regulation of initiation and maintenance phases of Hoxa3 expression in the vertebrate hindbrain involve auto- and cross-regulatory mechanisms. Development.

[bb0050] Marty T., Vigano M.A., Ribeiro C., Nussbaumer U., Grieder N.C., Affolter M. (2001). A HOX complex, a repressor element and a 50 bp sequence confer regional specificity to a DPP-responsive enhancer. Development.

[bb0055] Muller J., Thuringer F., Biggin M., Zust B., Bienz M. (1989). Coordinate action of a proximal homeoprotein binding site and a distal sequence confers the ultrabithorax expression pattern in the visceral mesoderm. EMBO J..

[bb0060] Noro B., Culi J., McKay D.J., Zhang W., Mann R.S. (2006). Distinct functions of homeodomain-containing and homeodomain-less isoforms encoded by homothorax. Genes Dev..

[bb0065] Packer A.I., Crotty D.A., Elwell V.A., Wolgemuth D.J. (1998). Expression of the murine Hoxa4 gene requires both autoregulation and a conserved retinoic acid response element. Development.

[bb0070] Pearson J.C., Lemons D., McGinnis W. (2005). Modulating Hox gene functions during animal body patterning. Nat. Rev. Genet..

[bb0075] Peifer M., Wieschaus E. (1990). Mutations in the drosophila gene extradenticle affect the way specific homeo domain proteins regulate segmental identity. Genes Dev..

[bb0080] Pinsonneault J., Florence B., Vaessin H., McGinnis W. (1997). A model for extradenticle function as a switch that changes HOX proteins from repressors to activators. EMBO J..

[bb0085] Popperl H., Bienz M., Studer M., Chan S.K., Aparicio S., Brenner S., Mann R.S., Krumlauf R. (1995). Segmental expression of hoxb-1 is controlled by a highly conserved autoregulatory loop dependent upon exd/pbx. Cell.

[bb0090] Rieckhof G.E., Casares F., Ryoo H.D., Abu-Shaar M., Mann R.S. (1997). Nuclear translocation of extradenticle requires homothorax, which encodes an extradenticle-related homeodomain protein. Cell.

[bb0095] Tremml G., Bienz M. (1992). Induction of labial expression in the *Drosophila* endoderm: response elements for dpp signalling and for autoregulation. Development.

[bb0100] Yau T.O., Kwan C.T., Jakt L.M., Stallwood N., Cordes S., Sham M.H. (2002). Auto/cross-regulation of Hoxb3 expression in posterior hindbrain and spinal cord. Dev. Biol..

[bb0105] Zabidi M.A., Arnold C.D., Schernhuber K., Pagani M., Rath M., Frank O., Stark A. (2015). Enhancer-core-promoter specificity separates developmental and housekeeping gene regulation. Nature.

[bb0110] Zappavigna V., Renucci A., Izpisua-Belmonte J.C., Urier G., Peschle C., Duboule D. (1991). HOX4 genes encode transcription factors with potential auto- and cross-regulatory capacities. EMBO J..

[bb0115] Zeng C., Pinsonneault J., Gellon G., McGinnis N., McGinnis W. (1994). Deformed protein binding sites and cofactor binding sites are required for the function of a small segment-specific regulatory element in Drosophila embryos. EMBO J..

